# Estimating epidemiological and economic burden and community derived disability weights for snake bite in Kerala: a study protocol

**DOI:** 10.12688/f1000research.50970.1

**Published:** 2021-03-01

**Authors:** Jaideep C. Menon, Denny John, Geeta R. Menon, Joseph K. Joseph, P. Rakesh Suseela, VV Pillay

**Affiliations:** 1 Cardiology, Amrita Institute of Medical Sciences, Amrita Vishwa Vidyapeetham, Kochi, Kerala, 682041, India; 2 Public health, Amrita Institute of Medical Sciences, Amrita Vishwa Vidyapeetham, Kochi, Kerala, 682041, India; 3 Biostatistics, National Institute of Medical Statistics, Indian Council of Medical Research, New Delhi, Delhi, 110029, India; 4 Nephrology, Little Flower hospital and Research centre, Angamaly, Kerala, 683572, India; 5 Poison Care Centre, Amrita Institute of Medical Sciences, Amrita Vishwa Vidyapeetham, Kochi, Kerala, 682041, India

**Keywords:** snakebite, epidemiology, economic burden, disability weight, DALY

## Abstract

**Background: **In India, geographical variation, lack of data and underreporting on cases and deaths due to snakebite makes it difficult to estimate socio-economic burden of snakebites. Previous studies measuring economic burden of snakebite in low-and-middle-income countries (LMICs) using different approaches have been conducted, but none so far in India. The proposed study aims to provide evidence on disability weights, epidemiological and economic burden due to snakebites in Kerala state, India.

**Protocol: **A cross-sectional community based study for estimating epidemiological and economic burden of snakebite, recruiting victims of snakebite occurring over a nine month period prior to start and over the three month period of the study, across Ernakulam district, Kerala state, India. For the community derived disability weights, 60 adult patients admitted and treated at Amrita Institute of Medical Sciences, Kochi or Little Flower Hospital, Angamaly would be interviewed. The sample size to determine the mortality rate is calculated at 108,458 persons in Ernakulam.The study will measure annual incidence, mortality, treatment cost of snakebites along with community-derived disability weights for snakebites. Standard methods for analysis and reporting of mortality, morbidity, years of lives lost, years lived with disability, disability weights, and costs of treatment will be calculated and presented. The study will be started in March 2021 and is expected to be completed by June 2021.

**Discussion**: This protocol is the first published for estimating epidemiological, economic burden and community derived disability weights for snakebites in India. Besides, the Global Burden of Disease has not attached a particular disability weight to snakebite and this would be an attempt to do so.The protocol has been developed using guidelines for both cross-sectional studies and  for conducting community derived disability weights. The evidence generated will contribute to knowledge regarding epidemiology, economic burden and community-derived disability weights for snakebites in India and other LMICs.

## Abbreviations

ASHA; Accredited Social Health Activist

CMO: Chief Medical Officer

DALY: Disability-adjusted life years

DW: Disability Weights

DMO: District Medical Officer

DPM: District Programme Management

GBD: Global Burden of Disease

NHM: National Health Mission

VAS: Visual Analogue Scale

YLD: Years lived with disability

YLL: Years of lives lost

## Introduction

Snakebite is a major public health problem in the rural communities of Asia, Africa, and Latin America, and most neglected among all the neglected tropical diseases. Many studies on the bio-medical perspectives of snakebite exist but very few studies have been conducted from a socio-economic viewpoint ^1^. Global estimates ofsnakebite range from 4.5 million to 5.4 million annually with an estimated 2 million of them in India, with tremendous socioeconomic consequences ^2^. As per the Registrar General of India-Million Death Study (RGI-MDS), the number of deaths due to venomous snakebite in India is 46,900 per year ^3^. Reports suggest that only 20–30% victims of snakebite in rural India seek treatment in hospitals ^4^. A recent update of the MDS suggests that the number of deaths are higher still at 58,000 per year ^5^.

The geographical variation, lack of data and underreporting on cases of snakebites and deaths makeit difficult to estimate socio-economic burden of snakebite in India. Few studies have provided data on mortality, cause of death , hospital based case series (in Maharashtra, West Bengal, Kerala and Andhra Pradesh states), compensations paid to snakebite victims and socio-economic impact ^6– 14^.

Along with mortality, snakebite may lead to physical and psychological impairment, scarring, permanent residual disability, blindness, malignant ulcers, pregnancy loss and of productivity following hospitalisation and incapacitation ^15^. The Disability Adjusted-Life Years (DALYs) is a widely used metric for quantifying disease burden ^16^. One DALY is equal to one lost year of healthy life. The sum of the DALYs for all diseases, across all age groups and either gender is a measure of the gap between current health status and an ideal health situation where the entire population lives to an advanced age, free of disease and disability.

Previous studies measuring economic burden of snakebites in low and middle income countries have used different approaches for estimating DALYs for snakebites. Kasturiratne *et al*. (2017), in measuring economic burden of snakebites in Sri Lanka, used disability weights for poisoning from the 2013 Global Burden of Disease (GBD) study as a surrogate ^17^. The duration of an episode of snakebite with envenoming was considered to be 0.3 years. For snakebite without envenoming disability weight of 0.006 (lower estimate) and 0.108 (higher estimate) were used; the higher estimate being a surrogate for open wounds as per the GBD study. The duration of illness of snakebite without envenoming was considered to be 0.04 years. Habib *et al*. (2015) used a meta-analytic approach to project annual epidemiological burden of snakebite envenoming in sub-Saharan Africa using pooled rates of incidence, amputation, and mortality rates ^18^. These estimates were applied to sub-Saharan population for deriving estimates of mortality and amputations. The standard loss functions based on projected frontier period life expectancy at birth for Japan and South Africa in the year 2050 estimated at 91.9 years (undiscounted) minus the mean age at the time of envenoming was used to calculate years of lives lost (YLL). Years lived with disability (YLD) were estimated by multiplying the number of amputations by the respective disability weight of 0.13 and applying this disability weight for the remainder of undiscounted local life expectancy. In Nigeria, Habib *et al*. (2015) used cost per DALY averted to measure the cost-effectiveness of antivenoms for snakebite envenoming ^19^. The study used associated amputation-related disability weight of 0.12.

This proposed study aims to address some of these issues by conducting a retrospective incidence study to provide evidence on disability weights, epidemiological and socio-economic burden due to snakebites in Kerala state, India.

The study will be conducted in Ernakulam district of Kerala to provide a state specific estimate on incidence, mortality, pattern of injuries, treatment seeking behaviour and cost of illness among snakebite victims. Additionally, the study will conduct a health state valuation to account for societal perspectives in estimating disability weights for snakebites.

### Rationale for the study

There is lack of data and underreporting on cases of snakebites and deaths that makes it difficult to estimate socio-economic burden of snakebite in India. Disability-weights (DWs) are values obtained from an individual’s perception of health states. It is to be noted that Global Burden of Disease (GBD) disability weights are not universal in nature as social and cultural contexts of health states were not accounted for in the GBD process ^20^. This is mainly because the GBD valuers generally, were educated professionals either from medical or health fields and could easily participate in the cognitively demanding valuation methods ^20– 25^. Although of late GBD and numerous other studies started including the general population along with professionals as participants, these studies were unable to capture the community-level perception of individual health states thus eliciting over-or-under-estimation of health states ^20, 26– 31^.

It is envisaged that the derived DWs from this proposed studyalong with the epidemiological and economic burden estimations, would be useful for future researchers and policymakers in the country to guide further research and policy in management of snakebites in the country.

### Aim and objectives

The aim of this study is to estimate the epidemiological and socio-economic burden and community-derived disability weights due to snakebites in Kerala state, India.

***Primary objectives***

1. To determine the prevalence, morbidity and mortality due to snakebite in Ernakulam district of Kerala state, India.

2. To determine the economic burden due to snakebites in the community.

3. To determine the community-derived disability weights for snakebites.

## Methods

### Study design

A cross-sectional study involving adult populations for estimating epidemiological and economic burden of snakebite will be conducted. These adults will be recruited over a total period of 12 months, 9 months prior to start of study and the 3 months of the study duration across various Gram Panchayats in Ernakulam district, Kerala state. For the community-derived disability weights, 60 adult patients admitted in the three months prior to start of data collection at either of Amrita Institute of Medical Sciences (AIMS), Kochi Hospital or Little Flowers Hospital (LF), Angamaly would be interviewed. The study will be started in March 2021 and is expected to be completed by June 2021.

### Study geography

Ernakulam district, with an area of 3063 sq. km, has a population of 3.47 million. Industry and service sectors are the main sources of occupation in the urban areas with agriculture being so in the eastern part of the district. Export oriented fishing industry is also a major source of revenue and occupation towards the coast.

### Study participants

***Epidemiological and economic burden*.** Victims and family members, identified by community health workers (ASHAs), with a history of snakebite in the preceding 12 months will be included for the socio-demographic and economic costsaspect of the study.

***Community-derived disability weights*.** The victims interviewed would include individuals who received treatment for snakebite either as an out or inpatient in the immediate nine months preceding the date of start of the study. The victims would include those identified at the community level by ASHA workers in addition to patients admitted and treated for snakebite within the three months of study duration in hospitals in the district treating snakebite. Victims thus identified from in-patient records of treating hospitals would be included, if residents of Ernakulam district. The length of hospital stay of individual patients would be accessed from both hospital records of patients admitted within the three month period of study and copies of discharge records of victims identified from community screening by ASHA workers.

For our study we will be using the Visual Analogue Scale (VAS) to valuate health states on a continuous graduated line segment, one end labelled as ‘death’ and the other labelled as ‘perfect health’ ranging from 0 to 100. The VAS allows the user to rate a particular health state between the mentioned anchor points, i.e. death and perfect health.

For the study component related to disability weights, victims admitted and treated for snakebite at AIMS, Kochi or Little Flower Hospital, Angamaly over the three month period prior to the start of study would be interviewed. Additionally, victims with a history of snakebite in the preceding nine months would also be interviewed for the epidemiologic and economic burden components of the study and prospectively so in victims admitted in the two afore-mentioned hospitals over the study period of three months.

Symptoms related to poisoning other than due to snakebite or non-ophid bites and those not willing to provide consent will be excluded.

### Sample size calculation

***Epidemiological and economic burden*.** For estimating the epidemiological and economic burden, a population-level epidemiological study will be conducted covering all gram panchayats (n= 82) in Ernakulam district of Kerala state, India, using a pre-specified questionnaire to capture demographic characteristics (area of residence, age, gender, education, household income etc), details of snakebite (envenomation, site, wound type), treatment (hospitalisation, outpatient, investigations), outcomes (number of days of hospitalisation, death) and costs (outpatient, investigations, hospitalisation, funeral). Based on the estimated population and households in a previous Sri Lankan study ^32^, taking the prevalence of snakebite as 153 per 100,000 population (0.15%), the estimated sample size is 5868 with a precision of 0.1% from a population of 3.47 million residents ^32^. Assuming 15% cases being unreported, a total of 6904 will need to be interviewed to identify 11 cases of snakebites. Since the mortality of snakebites is estimated at 6/100,000, the sample size to estimate a mortality of 0.006% is 92,190 persons. Assuming a 15% loss of information the actual sample size, to determine the mortality rate is 108,458 persons in Ernakulam.

$$\text{n}=\frac{\left(Z^{2})\text{P}(1-\text{P}\right)}{{\text{d}}^{\text{2}}}$$

Z _1-a/2_ = Is standard normal variate [at 5% type 1 error ( *P*<0.05) it is 1.96]. p = Expected proportion in population based on previous studies or pilot studies.

d = Absolute error or precision

***Community derived disability weights*.** The interviews will be conducted using a purposive sampling method and the VAS would be administered in about 60 adults currently admitted in AIMS and LF hospitals or admitted three months prior to data collection.

### Outcomes

The study will measure annual incidence, mortality, and treatment costs of snakebites in Ernakulam district of Kerala state, India. Additionally, the study will also calculate community-derived disability weights for snakebites in the district.

### Statistical analysis

***Epidemiological components*.** Population based incidence rates will be calculated using the “Survey” package in R programming language. Individual level variables (e.g. age, sex) will be considered only for descriptive analysis. The explanatory variables for snakebite incidence will include population density, sex, occupation, education, and income. The categorical variables will be presented in the form of frequencies and percentages and the continuous variables will be presented as means and standard deviations.

$$\text{Incidence}\;\text{risk}\;\text{=}\;\frac{\text{Number}\;\text{of}\;\text{incident}\;\text{cases}\;\text{of}\;\text{snakebites}\;\text{in}\;\text{the}\;\text{time}\;\text{period}\;\text{X}\;\text{100,000}}{\text{Population}\;\text{at}\;\text{risk}}$$

$$\text{Mortality}\;\text{risk}\;\text{=}\;\frac{\text{Number}\;\text{of}\;\text{deaths}\;\text{in}\;\text{the}\;\text{time}\;\text{period}\;\text{X}\;\text{100,000}}{\text{Population}\;\text{at}\;\text{risk}}$$

$$\text{Case-fatality}\;\text{rate}\text{=}\;\frac{\text{Number}\;\text{of}\;\text{deaths}\;\text{from}\;\text{snakebites}\;\text{in}\;\text{the}\;\text{time}\;\text{period}\;\text{X}\;\text{100,000}}{\text{Number}\;\text{of}\;\text{new}\;\text{cases}\;\text{of}\;\text{snakebites}\;\text{in}\;\text{the}\;\text{time}\;\text{period}}\;$$

***Cost of treatment*.** The median out-of-pocket cost of different cost elements (direct medical and non-medical and indirect) will be estimated based on the data reported by the victims or a household member.

***Health state valuation*.** For the health valuation descriptive statistics of the socio-demographic profile of the valuers will be presented using appropriate summary statistics—number with percentage for categorical variables and median with inter-quartile range for quantitative variables. The mean of the VAS scores for each disease sequelae will be calculated. The computation of DWs will be done using the formula: DW = 1 –VAS /100 ^33^. 95% Confidence Intervals will be provided for the DWs.

***Disability adjusted life years (DALYs)*.** DALYs for a disease or health condition is a combined metric of mortality and morbidity/disability and can be used to compare the disease burden across different countries or across different time periods for the same country. The mortality component is estimated in terms of YLL due to premature mortality, and the morbidity component is defined by the YLD due to that condition or any of its sequelae. DALYs is the sum of YLLs + YLDs ^34^. All the three metrics are defined for a particular health condition, for a pre-specified population whose age and sex wise population distribution, death/ mortality distribution, cause specific mortality distribution and life expectancy is known. The YLLs are then computed as the sum over all ages of the product of number of deaths at a particular age multiplied by the standard life expectancy at that age $\left(\text{YLL=}\sum_{x=\text{1}}^{n}{\text{N}}_{\text{x}}{\text{L}}_{\text{x}}\right)$ where N _x_= number of deaths at age x, L _x_= standard life expectancy in years at age x and x varies from 0 to n where n is the maximum years for which the population death data is available. YLDs for a particular cause (e.g. snakebite) in a particular time period, is calculated using the number of incident cases in that period multiplied by the average duration of the disease and the weight factor that reflects the severity of the disease from scale from 0 (perfect health) to 1 (death).

YLDs will be calculated using the incidence approach where YLD=I × DW × L, where I = number of incident cases, DW= disability weight, L= average duration of the case until recovery or death (years).

### Data collection, quality checks and monitoring

The study would be facilitated through the offices of the District Program Manager, National Health Mission and the Chief/District Medical Officer of Ernakulam district.

Informed consent from all participants will be conducted prior to administration of any data collection questionnaire ( *Extended data:* Annexure 1 ^35^). A pre-specified questionnaire developed for the research study will be used to collect data regarding socio-demographic, hospitalisation, and economic details ( *Extended data:* Annexure 2 ^35^). The questionnaire has been developed after consultation with experts working in the field of snakebites in the country, and discussion among authors. The interview schedule for valuation will have two sections: (1) socio-demographic profile of valuer, (2) ‘own health state’ valuation using VAS ( *Extended data:* Annexure 3 ^35^). Both these schedules will be pre-tested among 5-6 participants prior to any finalisation and administration to the entire sample.

However, for information related to any family member whose age is below 18 years, the mother or the father shall be interviewed. Information about the victim, profile of envenomation and complications thereof, other related characteristics, treatment outcome and any other related details will be noted but kept coded and confidential.

### Data entry and storage

Data entry will be conducted by a single data entry operator atthe research unit of AIMS. One of the co-authors will review the data entry to check for any discrepancies including any data entry errors from the data entry form. The data will be stored in a desktop computer with access to the data entry operator, and Principal Investigator (JCM). Once the data entry is completed and cleaned, the data sheet will be transferred to the laptops of the co-authors (JCM, GRM & DJ) for further analysis. After analysis these data sheets will be destroyed in these laptops and the data sheet would be available only with the desktop present at research unit of AIMS.

Data gathered at the Panchayat (village) level would be collated on a Tab PC by the field officers from where it would be synced on to the server at AIMS, accessible only to JCM,DJ and GRM. De-identified details would be used for statistical analysis and reporting. Details gathered would not be shared on any public domain and would be kept confidential.

## Ethical approval

Participants will be informed about the nature of the study and will be assured that privacy will be maintained, and information provided by the respondent will be held confidentially and only be used for research purposes. Their willingness to participate will be sought and informed written consent will be taken before including them in the study. Social and cultural values of the participants will be respected and considered as needed. Information obtained during research will not be used for any other purpose except research and research findings will be disseminated as per research dissemination ethics.

The study received ethics approval from the Institutional Ethics Committee of Amrita Institute of Medical Sciences, Kochi (study reference number IRB-AIMS-2020-1 01) on 13/03/2020.

## Study challenges

Snakebite is generally a disease of the working community, being most common among farmers, rubber tappers, tea/coffee estate pickers, brick kiln workers, and plywood industry workers. The majority of bites are accidents, which occur at the workplace or at home with the lower socio-economic groups being most affected. The degree of education and comprehension of the VAS could be a challenge in this group of individuals. There could be a recall bias in victims bitten close to a year back on degree of disability and other disease details as well.

## Distribution of study results

The study results will be submitted to a suitable peer-review publication within 6#six months of study completion. Additionally, the results will also be presented in suitable national/international conferences based on resources available for participation. The study results will be presented using STROBE guidelines for cross-sectional studies.

## Study status

The study protocol was discussed and agreed by Steering Group members (clinicians from Amrita Institute of Medical Sciences (AIMS), Kochi Hospital or Little Flowers Hospital (LF), Angamaly handling patients affected with snakebite) prior to start of data collection.

Training session of staff who would be administering the VAS score has been completed and the necessary permission for using frontline health workers in identifying victims of snakebite in the community has been secured from the District Program Manager of the National Health Mission’s office, and we expect to start the field work form the 1 ^st^ March 2021.

## Conclusion

This research constitutes the first published protocol for estimating epidemiological and economic burden as also community derived disability weights. The protocol has been developed using guidelines for cross-sectional studies, and international guidelines for conducting community-derived disability weights. The findings of the study will be useful to inform researchers for a proposed extension of the study in other states as part of ICMR-funded study to be initiated in 2021 (five of the authors are also investigators on this study). The evidence generated by this study will contribute significantly to knowledge regarding the epidemiology, economic burdenand community-derived disability weights for snakebite in India and other countries where incidence of snakebite is high.

## Data availability

### Underlying data

No underlying data is associated with this article.

### Extended data

Figshare: Estimating epidemiological and economic burden and community derived disability weights for snakebite in Kerala: A study protocol, https://doi.org/10.6084/m9.figshare.14061215.v1 ^35^

This project contains the following extended data:

- **Annexure 1.** Consent Form
- **Annexure 2.** Questionnaire
- **Annexure 3.** VAS tool

Data are available under the terms of the Creative Commons Attribution 4.0 International license (CC-BY 4.0).
