# Estimating epidemiological and economic burden and community derived disability weights for snake bite in Kerala: a study protocol

**DOI:** 10.12688/f1000research.50970.2

**Published:** 2021-06-11

**Authors:** Jaideep C. Menon, Denny John, Geeta R. Menon, Joseph K. Joseph, P. Rakesh Suseela, VV Pillay, Amitava Banerjee

**Affiliations:** 1 Cardiology, Amrita Institute of Medical Sciences, Amrita Vishwa Vidyapeetham, Kochi, Kerala, 682041, India; 2 Public health, Amrita Institute of Medical Sciences, Amrita Vishwa Vidyapeetham, Kochi, Kerala, 682041, India; 3 Biostatistics, National Institute of Medical Statistics, Indian Council of Medical Research, New Delhi, Delhi, 110029, India; 4 Nephrology, Little Flower hospital and Research centre, Angamaly, Kerala, 683572, India; 5 Poison Care Centre, Amrita Institute of Medical Sciences, Amrita Vishwa Vidyapeetham, Kochi, Kerala, 682041, India; 6 Institute of Health Informatics, University College London, London, NW1 2DA, UK

**Keywords:** snakebite, epidemiology, economic burden, disability weight, DALY

## Abstract

**Background: **In India, lack of data and underreporting of cases and deaths due to snakebite makes it difficult to estimate its socio-economic burden. Previous studies measuring economic burden of snakebite in low-and-middle-income countries (LMICs) using different approaches have been conducted, but none  in India. The proposed study aims to provide evidence on disability weights, epidemiological and economic burden due to snakebites in Kerala state, India.

**Protocol: **The study is a community based cross-sectional study recruiting victims of snakebite occurring over a 12 month period prior to start of the study , across  Ernakulam district, Kerala state, India. For the community-derived disability weights,70 adult patients who were treated within a 3 month period prior to commencement of the study at Amrita Institute of Medical Sciences, Kochi or Little Flower Hospital, Angamaly would be interviewed. The study will measure annual incidence, mortality, treatment cost of snakebites along with community-derived disability weights for snakebites in Ernakulam district.. Standard methods for analysis and reporting of mortality, morbidity, Years of Lives Lost (YLL), Years lived with disability (YLD), disability weights, and costs of treatment will be calculated. The study will be started in April 2021 and is expected to be completed by July2021..

**Discussion**: This protocol is the first published for estimating epidemiological, economic burden and community derived disability weights for snakebites in India. Besides, the Global Burden of Disease has not attached a particular disability weight to snakebite and this would be an attempt to do so. The protocol has been developed using guidelines for cross-sectional studies, cost of illness studies and international guidelines for conducting community derived disability weights. The evidence generated by this study will contribute significantly to knowledge regarding the epidemiology, economic burden and community-derived disability weights for snakebites in India and other countries where incidence of snakebite is high.

## Abbreviations

ASHA; Accredited Social Health Activist

CMO: Chief Medical Officer

DALY: Disability-adjusted life years

DW: Disability Weights

DMO: District Medical Officer

DPM: District Programme Management

EQ-5D: EuroQoL5 Dimension

GBD: Global Burden of Disease

NHM: National Health Mission

VAS: Visual Analogue Scale

YLD: Years lived with disability

YLL: Years of lives lost

## Introduction

Snakebite is a major public health problem in the rural communities of Asia, Africa, and Latin America, and most neglected among all the neglected tropical diseases. Many studies on the bio-medical perspectives of snakebite exist but very few studies have been conducted from a socio-economic viewpoint ^1^. Global estimates of snakebite range from 4.5 million to 5.4 million annually with an estimated 2 million of them in India, with tremendous socioeconomic consequences ^2^. As per the Registrar General of India-Million Death Study (RGI-MDS), the number of deaths due to venomous snakebite in India is 46,900 per year ^3^. Reports suggest that only 20–30% victims of snakebite in rural India seek treatment in hospitals ^4^. A recent update of the MDS suggests that the number of deaths are higher still at 58,000 per year ^5^.

The geographical variation, lack of data and underreporting on cases of snakebites and deaths make it difficult to estimate socio-economic burden of snakebite in India. Few studies have provided data on mortality, cause of death , hospital based case series (in Maharashtra, West Bengal, Kerala and Andhra Pradesh states), compensations paid to snakebite victims and socio-economic impact ^6– 14^.

Along with mortality, snakebite may lead to physical and psychological impairment, scarring, permanent residual disability, blindness, malignant ulcers, pregnancy loss and of productivity following hospitalisation and incapacitation ^15^. The Disability Adjusted-Life Years (DALYs) is a widely used metric for quantifying disease burden ^16^. One DALY is equal to one lost year of healthy life. The sum of the DALYs for all diseases, across all age groups and either gender is a measure of the gap between current health status and an ideal health situation where the entire population lives to an advanced age, free of disease and disability.

Previous studies measuring economic burden of snakebites in low and middle income countries have used different approaches for estimating DALYs for snakebites. Kasturiratne *et al*. (2017), in measuring economic burden of snakebites in Sri Lanka, used disability weights for poisoning from the 2013 Global Burden of Disease (GBD) study as a surrogate ^17^. The duration of an episode of snakebite with envenoming was considered to be 0.3 years. For snakebite without envenoming disability weight of 0.006 (lower estimate) and 0.108 (higher estimate) were used; the higher estimate being a surrogate for open wounds as per the GBD study. The duration of illness of snakebite without envenoming was considered to be 0.04 years. Habib *et al*. (2015) used a meta-analytic approach to project annual epidemiological burden of snakebite envenoming in sub-Saharan Africa using pooled rates of incidence, amputation, and mortality rates ^18^. These estimates were applied to sub-Saharan population for deriving estimates of mortality and amputations. The standard loss functions based on projected frontier period life expectancy at birth for Japan and South Africa in the year 2050 estimated at 91.9 years (undiscounted) minus the mean age at the time of envenoming was used to calculate years of lives lost (YLL). Years lived with disability (YLD) were estimated by multiplying the number of amputations by the respective disability weight of 0.13 and applying this disability weight for the remainder of undiscounted local life expectancy. In Nigeria, Habib *et al*. (2015) used cost per DALY averted to measure the cost-effectiveness of antivenoms for snakebite envenoming ^19^. The study used associated amputation-related disability weight of 0.12.

This proposed study aims to address some of these issues by conducting a retrospective incidence study to provide evidence on disability weights, epidemiological and socio-economic burden due to snakebites in Kerala state, India. The study will be conducted in Ernakulam district of Kerala to provide a state specific estimate on incidence, mortality, pattern of injuries, treatment seeking behaviour and cost of illness among snakebite victims. Additionally, the study will conduct a health state valuation to account for community perspectives in estimating disability weights for snakebites.

### Rationale for the study

There is lack of data and underreporting on cases of snakebites and deaths that makes it difficult to estimate socio-economic burden of snakebite in India. Disability-weights (DWs) are values obtained from an individual’s perception of health states. It is to be noted that Global Burden of Disease (GBD) disability weights are not universal in nature as social and cultural contexts of health states were not accounted for in the GBD process ^20^. This is mainly because the GBD valuers generally, were educated professionals either from medical or health fields and could easily participate in the cognitively demanding valuation methods ^20– 25^. Although of late GBD and numerous other studies started including the general population along with professionals as participants, these studies were unable to capture the community-level perception of individual health states thus eliciting over-or-under-estimation of health states ^20, 26– 31^.

It is envisaged that the derived DWs from this proposed study along with the epidemiological and economic burden estimations, would be useful for future researchers and policymakers in the country to guide further research and policy in management of snakebites in the country.

### Aim and objectives

The aim of this study is to estimate the epidemiological and socio-economic burden and community-derived disability weights due to snakebites in Kerala state, India.

***Primary objectives***

1. To determine the prevalence, morbidity and mortality due to snakebite in Ernakulam district of Kerala state, India.

2. To determine the economic burden due to snakebites in the community.

3. To determine the community-derived disability weights for snakebites.

## Methods

### Study design

**Figure 1.**
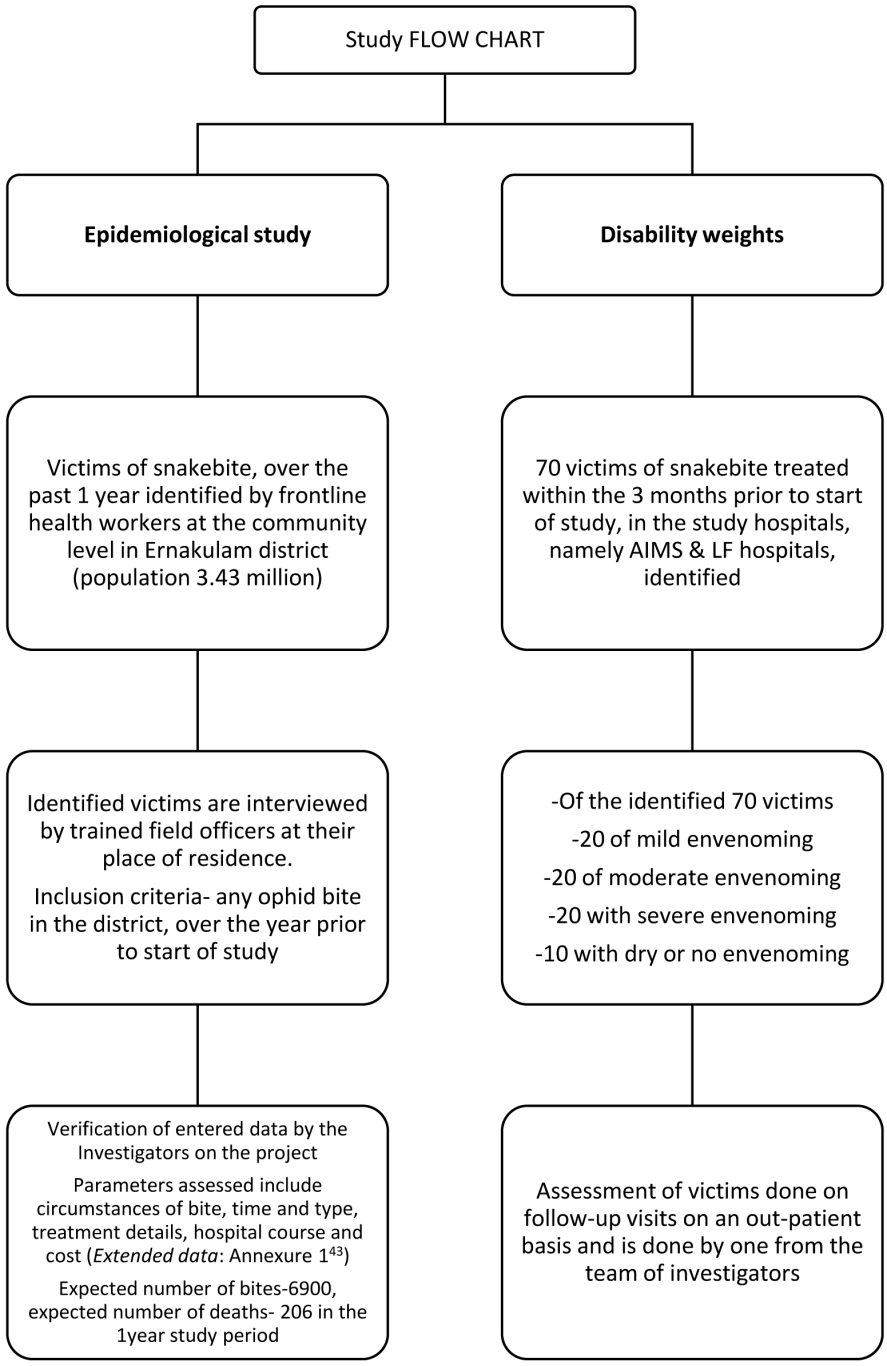
Patient inclusion flow chart for the epidemiologic and disability weight sub-sets of the study.

A cross-sectional study involving adult populations for estimating epidemiological and economic burden of snakebite will be conducted. These adults will be recruited over a total period of 12 months, prior to start of study across various Gram Panchayats in Ernakulam district, Kerala state. For the community-derived disability weights, 70 adult patients admitted in the three months prior to start of data collection at either of Amrita Institute of Medical Sciences (AIMS), Kochi Hospital or Little Flowers Hospital (LF), Angamaly would be interviewed. ( Figure 1) The study will be started in March 2021 and is expected to be completed by June 2021.

### Study geography

Ernakulam district, with an area of 3063 sq. km, has a population of 3.47 million. Industry and service sectors are the main sources of occupation in the urban areas with agriculture being so in the eastern part of the district. Export oriented fishing industry is also a major source of revenue and occupation towards the coast.

### Study participants

***Epidemiological and economic burden*.** Victims and family members, identified by community health workers (ASHAs), with a history of snakebite in the preceding 12 months will be included for the socio-demographic and economic costs aspect of the study.

***Community-derived disability weights*.** The victims interviewed would include individuals who received treatment for snakebite either as an out or inpatient in the immediate nine months preceding the date of start of the study. The victims would include those identified at the community level by ASHA workers in addition to patients admitted and treated for snakebite within the three months of study duration in hospitals in the district treating snakebite. Victims thus identified from in-patient records of treating hospitals would be included, if residents of Ernakulam district. The length of hospital stay of individual patients would be accessed from both hospital records of patients admitted within the three month period of study and copies of discharge records of victims identified from community screening by ASHA workers.

***Vignettes and 6D3L description system*.** Health states with description of symptoms along with functional statues are highly effective in health state valuation studies ^32, 33^. Along with the preparation of health state vignettes, a modified EQ-5D+ (EuroQoL) instrument will be used alongside the vignettes to further describe the valuer’s functional status. In order to ensure the understanding of long, complicated sentences (vignettes) among the valuers in the sample population (which are mainly rural and semi-literature), a reduced version of 5 dimensions of EQ-5D to 3 dimensions that has been previously validated in India will be used ^33^. Table 1 provides the 6D3L description system.

**Table 1.** 6D-3L description system.

Dimension	Dimension description	Severity level
Mobility	Getting around in the community, walking, climbing stairs, etc.	1- No problems walking about 2- Some problems walking about 3- Confined to bed
Self-care	Bathing, cleaning, washing, toileting et.	1- No problems with self-care 2- Some problems washing or dressing self 3- Unable to wash or dress self
Usual activity	Performance of usual role activities such as working at a job, housework, childcare, volunteer work, etc.	1- No problems with performing usual activities 2- Some problems with performing usual activities 3- Unable to perform usual activities
Pain/Discomfort	Subjective feeling of bodily distress of discomfort	1- No pain or discomfort 2- Moderate pain or discomfort 3- Extreme pain or discomfort
Anxiety/depression	Negative psychological states including anxiety, depression, behavioural emotional control, loneliness, etc.	1- Not anxious or depressed 2- Moderately anxious or depressed (social isolation or loss of appetite) 3- Extremely anxious or depressed (suicidal ideation)
Cognition	Cognitive problems, such as forgetfulness, difficulty in concentrating, loss of tempero-spatial orientation, etc.	1- No problems in cognition 2- Some problem with memory and concentration 3- Severe problem in cognition (loss of tempero-spatial orientation)

The application of EQ-6D-3L will be conducted using a modified card sort (CS) method rather than the cognitively demanding techniques such as standard gamble (SG), time trade-off (TTO), and patient trade-off (PTO) that required certain level of education to comprehend and use for estimating disability weights ^34^. Any process of eliciting valuation of health states irrespective of the technique used requires valuers (i.e. the patient or care-giver) to visualize the entire description of health states. The modified CS process used prior to VAS will not only be a validation tool but also a “warm up” for the entire valuation process. 

We will be using a set of pictorial narrations describing the three severity levels under each of the six dimensions, i.e., 18 pictures will be prepared. The local social context relevant to the geographical and cultural settings are depicted through the pictures. Several drafts of pictures were drawn by a commercial artist and shared with the study team for final set of 18 pictures to be used (Figure available in *Extended data*: Annexure 3 ^35^).

To overcome the issue of ranking and assigning scores, the study team decided to divide the rank order into two parts for ease of understanding. Thus, during CS, the description of each health state was read out to the valuers in random order by the interviewer, who would then be instructed to rank their preference between 1 and 5 for less severe health states (according to their choice) and 6–11 for more severe.

For our study we will be using the Visual Analogue Scale (VAS) to valuate health states on a continuous graduated line segment, one end labelled as ‘death’ and the other labelled as ‘perfect health’ ranging from 0 to 100. The VAS allows the user to rate a particular health state between the mentioned anchor points, i.e. death and perfect health. A picture of a happy face near “100” on one side and a picture of a sad face near “0” will further help the valuer to the direction of severity (Extended data: Annexure 3 ^35^).

At the end of both exercises, the CS rank and VAS scores will be checked for concordance, by the investigator. In instances where the values and ranks did not correspond, the valuer will be requested to review his/her responses through iterations by the investigator. Multiple iterations would be conducted until the valuer’s response for each health state was final.

The interview schedule will have four sections: (1) socio-demographic profile of valuer; (2) “own health state” valuation using VAS; (3) Hospitalisation details, (4) EQ-6D-3L scores, and (5) VAS scores.

So as to account for victims with different degrees of severity of envenoming we shall interview twenty each victims of mild, moderate and severe envenoming and ten of non-venomous / dry bites. We shall go by the classification; **mild** envenoming as victims who had symptoms limited to local reaction at the bite site without signs and symptoms of systemic envenoming and or a hospital stay of < 3 days, **moderate** qualified as presence of signs and symptoms and laboratory parameters suggestive of systemic envenoming and or a hospital stay of 3–5 days and **severe** being qualified by any one of life threatening, hospital stay > 7 days, need for a surgical procedure, ventilation support, dialysis or requirement of blood products. Non-venomous/ dry bites are characterised by absence of signs and symptoms of either local or systemic envenoming. 

For the study component related to disability weights, victims admitted and treated for snakebite at AIMS, Kochi or Little Flower Hospital, Angamaly 3 months prior to the start of study would be interviewed. The main reason to choose admitted patients over the community based individuals is that the VAS and EQ-6D-3L being complex to administer will be conducted by JCM.

For the epidemiological and economic burden components of the study, victims with a history of snakebite in the preceding 12 months would be interviewed.

Symptoms related to poisoning other than due to snakebite or non-ophid bites and those not willing to provide consent will be excluded.

### Sample size calculation

***Epidemiological and economic burden*.** For estimating the epidemiological and economic burden, a population-level epidemiological study will be conducted covering all gram panchayats (n= 82) in Ernakulam district of Kerala state, India, using a pre-specified questionnaire to capture demographic characteristics (area of residence, age, gender, education, household income etc.), details of snakebite (envenomation, site, wound type), treatment (hospitalisation, outpatient, investigations), outcomes (number of days of hospitalisation, death) and costs (outpatient, investigations, hospitalisation, funeral). In a study from Sri Lanka, using the prevalence of snakebite as 153 per 100,000 population (0.15%), the estimated sample size was 5868 with a precision of 0.1% from a population of 3.47 million residents ^36, 37^. Assuming 15% cases being unreported a total of 6904 have to be covered to identify 11 cases of snakebites in a 100,000 population.

For our study, the annual mortality of snakebites is estimated at 6/100,000 ^3, 5^, and the sample size to estimate a mortality of 0.006 percent will be 92,190 persons. Assuming a 15 % loss of information the actual sample size to determine the mortality rate is 108,458 persons in Ernakulam (representing 3.18% of total population (3.4 million as per Census of India 2011 estimates) of Ernakalum district.

The following formula is used to estimate sample size for our study:

$$\text{n}=\frac{\left(Z^{2})\text{P}(1-\text{P}\right)}{{\text{d}}^{\text{2}}}$$

Z _1-a/2_ = Is standard normal variate [at 5% type 1 error ( *P*<0.05) it is 1.96]. p = Expected proportion in population based on previous studies or pilot studies.

d = Absolute error or precision

***Community derived disability weights*.** The interviews will be conducted using a purposive sampling method and the VAS would be administered in about 70 adults currently admitted in AIMS and LF hospitals or admitted three months prior to data collection.

### Outcomes

The study will measure annual incidence, mortality, and treatment costs of snakebites in Ernakulam district of Kerala state, India. Additionally, the study will also calculate community-derived disability weights for snakebites in the district.

### Statistical analysis

***Epidemiological components*.** Population based incidence rates will be calculated using the “Survey” package in R programming language. Individual level variables (e.g. age, sex) will be considered only for descriptive analysis. The explanatory variables for snakebite incidence will include population density, sex, occupation, education, and income. The categorical variables will be presented in the form of frequencies and percentages and the continuous variables will be presented as means and standard deviations.

$$\text{Incidence}\;\text{risk}\;\text{=}\;\frac{\text{Number}\;\text{of}\;\text{incident}\;\text{cases}\;\text{of}\;\text{snakebites}\;\text{in}\;\text{the}\;\text{time}\;\text{period}\;\text{X}\;\text{100,000}}{\text{Population}\;\text{at}\;\text{risk}}$$

$$\text{Mortality}\;\text{risk}\;\text{=}\;\frac{\text{Number}\;\text{of}\;\text{deaths}\;\text{in}\;\text{the}\;\text{time}\;\text{period}\;\text{X}\;\text{100,000}}{\text{Population}\;\text{at}\;\text{risk}}$$

$$\text{Case-fatality}\;\text{rate}\text{=}\;\frac{\text{Number}\;\text{of}\;\text{deaths}\;\text{from}\;\text{snakebites}\;\text{in}\;\text{the}\;\text{time}\;\text{period}\;\text{X}\;\text{100,000}}{\text{Number}\;\text{of}\;\text{new}\;\text{cases}\;\text{of}\;\text{snakebites}\;\text{in}\;\text{the}\;\text{time}\;\text{period}}\;$$

***Cost of treatment*.** The median out-of-pocket cost of different cost elements (direct medical and non-medical and indirect) will be estimated based on the data reported by the victims or a household member. We will use cost of treatment episode to calculate direct medical and non-medical costs. For indirect costs the number of days of work loss along with daily wage/total wage loss will be calculated ^38^.

***Health state valuation*.** For the health valuation descriptive statistics of the socio-demographic profile of the valuers will be presented using appropriate summary statistics—number with percentage for categorical variables and median with inter-quartile range for quantitative variables. The mean of the VAS scores for each disease sequelae will be calculated. The computation of DWs will be done using the formula: DW = 1 –VAS /100 ^33^. 95% Confidence Intervals will be provided for the DWs.

***Disability adjusted life years (DALYs)*.** DALYs for a disease or health condition is a combined metric of mortality and morbidity/disability and can be used to compare the disease burden across different countries or across different time periods for the same country. The mortality component is estimated in terms of YLL due to premature mortality, and the morbidity component is defined by the YLD due to that condition or any of its sequelae. DALYs is the sum of YLLs + YLDs ^34^. All the three metrics are defined for a particular health condition, for a pre-specified population whose age and sex wise population distribution, death/ mortality distribution, cause specific mortality distribution and life expectancy is known. The YLLs are then computed as the sum over all ages of the product of number of deaths at a particular age multiplied by the standard life expectancy at that age $\left(\text{YLL=}\sum_{x=\text{1}}^{n}{\text{N}}_{\text{x}}{\text{L}}_{\text{x}}\right)$ where N _x_= number of deaths at age x, L _x_= standard life expectancy in years at age x and x varies from 0 to n where n is the maximum years for which the population death data is available. YLDs for a particular cause (e.g. snakebite) in a particular time period, is calculated using the number of incident cases in that period multiplied by the average duration of the disease and the weight factor that reflects the severity of the disease from scale from 0 (perfect health) to 1 (death).

YLDs will be calculated using the incidence approach where YLD=I × DW × L, where I = number of incident cases, DW= disability weight, L= average duration of the case until recovery or death (years).

### Data collection, quality checks and monitoring

The study would be facilitated through the offices of the District Program Manager, National Health Mission and the Chief/District Medical Officer of Ernakulam district.

Trained ASHAs attached to each Gram Panchayat will inform 2 Field Officers specially recruited for the study of the families reporting snakebite episodes 365 days prior in a particular village. The field officers will then be responsible to administer the informed consent and study questionnaire ( *Extended data*: Annexure 1 ^35^).

From the hospital register of AIMS and LF contact details of patients admitted and discharged 3 months to survey period will be identified. These patients and/or care-givers (if patient had died post discharge) will be invited to visit AIMS and will be interviewed by JCM. JCM will also be responsible for extracting hospitalisation information from medical records from AIMS or LF of each interviewed patient/care-giver.

Data gathered at the Panchayat (village) level would be collated on a Tab PC by the field officers from where it would be synced on to the server at Amrita Institute of Medical Sciences, accessible only to JCM,DJ and GRM. De-identified details would be used for statistical analysis and reporting. Details gathered would not be shared on any public domain and would be kept confidential. Any data queries will be handled using a data access committee ^39^.

Informed consent from all participants will be conducted prior to administration of any data collection questionnaire ( *Extended data:* Annexure 1 ^35^). A pre-specified questionnaire developed for the research study will be used to collect data regarding socio-demographic, hospitalisation, and economic details ( *Extended data:* Annexure 2 ^35^). The questionnaire has been developed after consultation with experts working in the field of snakebites in the country, and discussion among authors. The interview schedule for valuation will have two sections: (1) socio-demographic profile of valuer, (2) ‘own health state’ valuation using VAS ( *Extended data:* Annexure 3 ^35^). Both these schedules will be pre-tested among 5-6 participants prior to any finalisation and administration to the entire sample.

However, for information related to any family member whose age is below 18 years, the mother or the father shall be interviewed. Information about the victim, profile of envenomation and complications thereof, other related characteristics, treatment outcome and any other related details will be noted but kept coded and confidential.

### Data entry and storage

Data entry will be conducted by a single data entry operator at the research unit of AIMS. One of the co-authors will review the data entry to check for any discrepancies including any data entry errors from the data entry form. The data will be stored in a desktop computer with access to the data entry operator, and Principal Investigator (JCM). Once the data entry is completed and cleaned, the data sheet will be transferred to the laptops of the co-authors (JCM, GRM & DJ) for further analysis. After analysis these data sheets will be destroyed in these laptops and the data sheet would be available only with the desktop present at research unit of AIMS.

Data gathered at the Panchayat (village) level would be collated on a Tab PC by the field officers from where it would be synced on to the server at AIMS, accessible only to JCM,DJ and GRM. De-identified details would be used for statistical analysis and reporting. Details gathered would not be shared on any public domain and would be kept confidential.

## Ethical approval

Participants will be informed about the nature of the study and will be assured that privacy will be maintained, and information provided by the respondent will be held confidentially and only be used for research purposes. Their willingness to participate will be sought and informed written consent in a language understood by the respondent (English or Malayalam) will be taken before including them in the study. For children an assent form will be used. Social and cultural values of the participants will be respected and considered as needed. Information obtained during research will not be used for any other purpose except research and research findings will be disseminated as per research dissemination ethics.

The study received ethics approval from the Institutional Ethics Committee of Amrita Institute of Medical Sciences, Kochi (study reference number IRB-AIMS-2020-1 01) on 13/03/2020.

## Study challenges

Snakebite is generally a disease of the working community, being most common among farmers, rubber tappers, tea/coffee estate pickers, brick kiln workers, and plywood industry workers. The majority of bites are accidents, which occur at the workplace or at home with the lower socio-economic groups being most affected. The degree of education and comprehension of the CS and VAS could be a challenge in this group of individuals. There could be a recall bias in victims bitten close to a year back on degree of disability and other disease details as well. Additionally, the community responses for healthcare expenditure could also be subject to overestimation due to self-reported recall bias.

## Distribution of study results

The study results will be submitted to a suitable peer-review publication within 6#six months of study completion. Additionally, the results will also be presented in suitable national/international conferences based on resources available for participation. The study results for epidemiology will be presented using STROBE guidelines for cross-sectional studies ^40^ and cost of illness checklist adapted from Larg and Moss (2011) ^41^.

## Study status

The study protocol was discussed and agreed by Steering Group members (clinicians from Amrita Institute of Medical Sciences (AIMS), Kochi Hospital or Little Flowers Hospital (LF), Angamaly handling patients affected with snakebite) prior to start of data collection.

Training session of staff who would be administering the VAS score has been completed and the necessary permission for using frontline health workers in identifying victims of snakebite in the community has been secured from the District Program Manager of the National Health Mission’s office, and we expect to start the field work form the 1 ^st^ April 2021.

## Discussion

Our study uses a community survey to estimate incidence of mortality, morbidity and disability and economic burden of snakebites in Ernakulam district along with use of Visual Analogue Scale (VAS) as the tool for obtaining values required for computing community-derived disability weights. We have used sample size as per standard calculations to estimate epidemiological and economic burden at district level. For the community-derived disability weights we will be using VAS which is a tested and validated method since the early 1990s. We have deliberately combined VAS with card sort method rather than the alternate PTO, TTO or SG, or recent paired comparisons (PC) or discrete choice experiments (DCE) to order to ensure easier comprehension by community members across different sections of society and location. Our method follows the standard protocol that has been tested previously in Indian settings ^34, 42, 43^. We also believe that this pilot initiative for estimating community derived disability weights for snakebite using cognitively less demanding tools on the values would be an appropriate choice for rural and less educated populations for understanding their health preferences in India and similar settings in other developing countries. 

As per our knowledge, this is the first methodological protocol developed for estimating epidemiological and economic burden along with community derived disability weights on snakebites in a LMIC setting. Our approaches described for each of the study components, i.e. epidemiology, economic, and health state valuation has been described in meticulous detail. We are hopeful that this well-designed, systematic protocol can be used across various locations, cultures and even countries to explore health state values for other diseases as well. Our method describing the community-derived disability weights will be useful to guide researchers to implement such studies at community level in the future.

## Conclusion

This paper constitutes the first published protocol for estimating epidemiological and economic burden as also community derived disability weights for snakebite in LMICs. The protocol has been developed using guidelines for cross-sectional studies, and international guidelines for conducting community-derived disability weights. The findings of the study will be useful to inform researchers for a proposed extension of the study in other states as part of ICMR-funded study to be initiated in 2021(five of the authors are also investigators on this study). The evidence generated by this study will contribute significantly to knowledge regarding the epidemiology, economic burden and community-derived disability weights for snakebite in India and other countries where incidence of snakebite is high, thus playing a significant role in policy decision making for resource allocation in snakebite prevention and healthcare provision.

## Data availability

### Underlying data

No underlying data is associated with this article.

### Extended data

Figshare: Estimating epidemiological and economic burden and community derived disability weights for snakebite in Kerala: A study protocol, https://doi.org/10.6084/m9.figshare.14061215.v3 ^35^

This project contains the following extended data:

- **Annexure 1.** Consent Form.docx
- **Annexure 2.** Questionnaire.docx
- **Annexure 3.** VAS tool.docx
- Vignettes for EQ-6D-3L.zip

Data are available under the terms of the Creative Commons Attribution 4.0 International license (CC-BY 4.0).
