# Supplementary material for: Estimating epidemiological and economic burden and community derived disability weights for snake bite in Kerala: a study protocol
Source: F1000Res. 2021 Jun 11;10:167. Originally published 2021 Mar 1. [Version 2] doi: 10.12688/f1000research.50970.2 (PMC8278250; doi:10.12688/f1000research.50970.2)
Supplement: Peer review history

# Peer review: Reviewer response for version 2

**DOI:** 10.5256/f1000research.57297.r87367

**Published:** 2021-07-13

**Authors:** Abul M. Faiz

**Affiliations:** 1 Scientist, Dev Care Foundation, Dhaka, Bangladesh

There is significant improvement of the document after incorporating comments and adding a new table, providing much clarity.

 Some additional points may be considered:

1. Introduction: In the last paragraph the sentence beginning with ‘The study will be...', may be put before the preceding sentence, 'This proposed study aims...’.
2. Aim and objectives: Separate sub-heading for aim may be provided, like 'primary objective' (which may be objective as there is no primary objective mentioned here).
3. There is still inconsistency that needs correction: Text mentioned only adult patients will be included, but the Ethical approval section mentioned: ‘For children an assent form will be used.’.
4. It may be clarified how 70 patients (and from each category 20+20+20+10) will be selected.
5. During this COVID-19 pandemic, assuming that public health measures will be ensured during the study, the use of PPE (e.g. masks), social distancing, and use of hand sanitizers may be mentioned in the text.

Is the study design appropriate for the research question?

Partly

Is the rationale for, and objectives of, the study clearly described?

Partly

Are sufficient details of the methods provided to allow replication by others?

No

Are the datasets clearly presented in a useable and accessible format?

Partly

Reviewer Expertise:

Clinician with special interest in snakebite, poisoning and infectious diseases

I confirm that I have read this submission and believe that I have an appropriate level of expertise to confirm that it is of an acceptable scientific standard.

# Peer review: Reviewer response for version 2

**DOI:** 10.5256/f1000research.57297.r87366

**Published:** 2021-06-25

**Authors:** Louis W Niessen

**Affiliations:** 1 Department of International Public Health, Liverpool School of Tropical Medicine, Liverpool, UK

- The major differences have been addressed appropriately and are more in line with the formal GBD method to compute DALYs.
- The addition of the Visual Analogue Scale (VAS) helps to clinically assess QoL after snake bites.
- The flow chart outlines in detail the patient inclusion and steps in the analyses, leading to a better picture of the epidemiology and economic burden as well as the DALY calculation.
- External validity is to be addressed and dealt with in careful terms.

Is the study design appropriate for the research question?

Yes

Is the rationale for, and objectives of, the study clearly described?

Yes

Are sufficient details of the methods provided to allow replication by others?

Partly

Are the datasets clearly presented in a useable and accessible format?

Not applicable

Reviewer Expertise:

Health economics and epidemiology in particular burden of disease methods and clinical studies.

I confirm that I have read this submission and believe that I have an appropriate level of expertise to confirm that it is of an acceptable scientific standard.

# Peer review: Reviewer response for version 1

**DOI:** 10.5256/f1000research.54070.r81325

**Published:** 2021-04-06

**Authors:** Louis W Niessen

**Affiliations:** 1 Department of International Public Health, Liverpool School of Tropical Medicine, Liverpool, UK

The study protocol describe the various sub-studies needed to estimate the health and economic burden from snakebite in Kerala, conform the global BOD approach. There are 1) hospital-based study including patients with a present snakebite or a history of snakebite, 2) a district level community study in all panchayats, target about 9000 inhabitants to identify and track people who had a recent snakebite. The study is very useful and timely for the India situation and globally in the NTD discussions. Some descriptions of some methods are not very clearly (see three points below). The additional minor points (see further below) can be addressed relatively easily. 

**To be addressed:**

1. I would recommend a patient inclusion flow diagram. The exact number of hospital patients to be included is not clear nor the expected number per panchayat. It is unclear how hospital patients with a history of snakebite are identified and included for an interview in addition to the 62 presenting patients. It is unclear if at the community level what the sample framework is (sample 108,458 or 6904 persons? Percentage of total panchayat population?). Will there be an attempt to identify the missing fatal cases in the past, through registries or verbal autopsies? Any limitations to recall bias to take into account?
2. A DALY approach is proposed, yet, in spite of the many DALY refs, it is unclear how conform this will be to the international (Seattle-based) standards, especially the way disability-weights are established, like is done in other countries, through professionals and, possibly, at community levels. Only a (clinical) VAS approach is proposed, which is used in QALYs computations, using EuroQol versions. Presently, this sounds like quite a mixed bag. Perhaps this can be added in a better narrative in a Box. Will there be controls i.e. health matches? How will the findings be validated / compared against the present BOD weights? Against other India studies?
3. How is snakebite short-term and long-term morbidity defined? In clinical terms? How are the VAS and clinical data reconciled, statistically? 

**Minor points**

- How are data queries handled?
- Is there a quality assurance process, collecting data at the community and/or at the hospital? How?
- Is the a specific pilot / testing protocol / field training on the use of VAS in illiterate participants?
- Can one add a scored STROBE check list, please?
- What are the challenges of generalisability? How are findings extrapolated at Kerala / all India level (as state 'country')?
- What kind of costing is taking place? Perspective? Time horizon? Which checklist is used? CHEERS?
- Some wording needs to be improved like in the Abstract: 'methods are calculated and presented'.
- Can the actual guidelines used for the BOD analysis and the community disability weights be added?
- Is EuroQol approval needed for use of the VAS?

Is the study design appropriate for the research question?

Yes

Is the rationale for, and objectives of, the study clearly described?

Yes

Are sufficient details of the methods provided to allow replication by others?

Partly

Are the datasets clearly presented in a useable and accessible format?

Not applicable

Reviewer Expertise:

health economics and epidemiology in particular burden of disease methods and clinical studies.

I confirm that I have read this submission and believe that I have an appropriate level of expertise to confirm that it is of an acceptable scientific standard, however I have significant reservations, as outlined above.

# Response

**Published:** 2021-05-27

**Authors:** Jaideep Menon

**Affiliations:** Amrita Institute of Medical Sciences, India

Dr Louis Niessen

 The study protocol describes the various sub-studies needed to estimate the health and economic burden from snakebite in Kerala, conform the global BOD approach. There are 1) hospital-based study including patients with a present snakebite or a history of snakebite, 2) a district level community study in all panchayats, target about 9000 inhabitants to identify and track people who had a recent snakebite. The study is very useful and timely for the India situation and globally in the NTD discussions. Some descriptions of some methods are not very clearly (see three points below). The additional minor points (see further below) can be addressed relatively easily.

 To be addressed:

 1.            I would recommend a patient inclusion flow diagram. The exact number of hospital patients to be included is not clear nor the expected number per panchayat. It is unclear how hospital patients with a history of snakebite are identified and included for an interview in addition to the 62 presenting patients. It is unclear if at the community level what the sample framework is (sample 108,458 or 6904 persons? Percentage of total panchayat population?). Will there be an attempt to identify the missing fatal cases in the past, through registries or verbal autopsies? Any limitations to recall bias to take into account?

 Response: We have now included a patient inclusion flow diagram to provide an overview of the patients included for the study. The hospitalised patients will be sub-set of the population survey however they will be subjected to EQ-6D-3L and VAS in addition to other details as mentioned in Annexure 3. The total sample in the population for the epidemiological component is 108,458 persons representing 3.18% of the total population of Ernakulam district. Our studies uses ASHAs who are attached to each Gram Panchayat to report the episodes related to snakebites, and has regular interaction with the community. Due to long collaborations of working with ASHAs by JCM in Ernakulam district it is assumed that ASHAs will have knowledge of snakebite victims in each Gram Panchayat based on questioning with villagers. We will not be able to corroborate any missing cases with registries as data for 2018 year on causes of death is only available in February 2021. We do not aim to use verbal autopsy methods as snakebite is an easily reported event due to the circumstantial and symptomatic evidence as gathered from  the community/family member as well as treating clinician.

 2.            A DALY approach is proposed, yet, in spite of the many DALY refs, it is unclear how conform this will be to the international (Seattle-based) standards, especially the way disability-weights are established, like is done in other countries, through professionals and, possibly, at community levels. Only a (clinical) VAS approach is proposed, which is used in QALYs computations, using EuroQol versions. Presently, this sounds like quite a mixed bag. Perhaps this can be added in a better narrative in a Box. Will there be controls i.e. health matches? How will the findings be validated / compared against the present BOD weights? Against other India studies?

 Response: We have revised the protocol to the use of EQ-6D-3L approach using modified card sorting method to visualise the entire description of health states. The modified CS process using EQ-6D-3L which has been validated in previous studies in Indian settings will not only act as a validation tool but also as a ‘warm-up” for the entire valuation exercises. Our use of EQ-6D-3L is is also due to the fact that other techniques such as Standard Gamble,TTO, PTO, patient elicitation and DCE are complicated to administer among rural and semi-literature populations. There are other studies that have used EQ-6D-3L (Lobo et.al. 2019; Mahpatra et. al., 2000) hence our findings will be validated against these studies published previously. We will not be using any healthy controls as this is not the focus of current study, and might be something to be conducted in future. We have also used pictorial diagrams to be used in vignettes of EQ-6D-3L component so that the valuers can visualize the entire description of health states.

 3.            How is snakebite short-term and long-term morbidity defined? In clinical terms? How are the VAS and clinical data reconciled, statistically?

 We are using 1-month period as short-term morbidity and long-term morbidity between 1-3 months duration. We will be capturing clinical data of patients on whom VAS will be administered and will use severity as per definition used in our study using standard statistical methods

 Minor points

 •             How are data queries handled?

 Any data queries will be handled by a data access committee, the details are now mentioned in the revised manuscript.

 •             Is there a quality assurance process, collecting data at the community and/or at the hospital? How?

 Quality checks of Annexure 1 will be conducted by JCM on 1% of total sampled patients to corroborate data collection collected by field officers. For hospitalised cases, JKJ or VVP will reconduct among sample of patients (5% of patients) for validation of responses. In case the differences of EQ-6D-3L or VAS scores are substantial then the choice of scores will be used based on discussions with JCM, JKJ or VVP and will be arrived through consensus.

  •            Is the a specific pilot / testing protocol / field training on the use of VAS in illiterate participants?

 Both the questionnaires used in the study will be pre-tested among 5-6 participants prior to any finalisation and administration to the entire sample.

 •             Can one add a scored STROBE check list, please?

 STROBE checklists are used for reporting of studies hence we have not provided scores as part of the protocol. We have also added the use of cost of illness checklist by Larg and Moss (2011) to report economic components of the study.

 •             What are the challenges of generalisability? How are findings extrapolated at Kerala / all India level (as state 'country')?

 Our study methods and findings will be adapted after any suitable modifications to the ICMR-funded national study that JCM is PI and other members are Co-PIs. We will also be repeating the community derived disability weights methods in other states for generalisability of findings from our study to other states.

  •            What kind of costing is taking place? Perspective? Time horizon? Which checklist is used? CHEERS?

 We will be using an expenditure method to derive cost of treatment and indirect cost to calculate economic costs (i.e. a prevalence approach of cost of illness method). This will be conducted using a societal perspective with a time horizon to a maximum of 12 months prior to data collection. We will be using a questionnaire to capture the resources used for treatment along with costs. Additionally costs of funeral and any subsidy through government insurance and any other means will also be captured. We will use cost of illness checklist by Larg and Moss (2011) to report our study findings.

 •             Some wording needs to be improved like in the Abstract: 'methods are calculated and presented'.

 Abstract has been revised suitably.

  •            Can the actual guidelines used for the BOD analysis and the community disability weights be added?

 We have mentioned use of Vignettes and EQ-6D-3L description system along with DW calculations mentioned. The methods for calculating DW mentioned in our study has been used in previous studies in Indian settings.

  •            Is EuroQol approval needed for use of the VAS?

 No.

 In addition Dr Amitava Banerjee (UCL) a long-term GBD collaborator in onboard the study team to help navigate the GBD part of the study.

 •             Is the rationale for, and objectives of, the study clearly described?

 Yes

 •             Is the study design appropriate for the research question?

 Yes

 •             Are sufficient details of the methods provided to allow replication by others?

 Partly

 •             Are the datasets clearly presented in a useable and accessible format?

 We have presented the datasets in an online repository available at https://doi.org/10.6084/m9.figshare.14061215.v1

 ________________________________________

 Competing Interests

 No competing interests were disclosed.

 ________________________________________

 Reviewer Expertise

 health economics and epidemiology in particular burden of disease methods and clinical studies.

# Peer review: Reviewer response for version 1

**DOI:** 10.5256/f1000research.54070.r81324

**Published:** 2021-03-23

**Authors:** Abul M. Faiz

**Affiliations:** 1 Scientist, Dev Care Foundation, Dhaka, Bangladesh

Snakebite is labelled as 'injuries' which is not entirely true. This research will be used for further research and policy formulation, instead may be stated as utilized for policy decision and further research.

 Title: aim and objectives may be mentioned/ changed as  'Objectives with division: General objective (s), and specific objectives.

 Being an emergency situation snakebite attending at OPD is unlikely and to be replaced by emergency department or in-patient department, also better to include any victims of snakebite.

 Study design: some contradictory statement are there: adults but mentioned that consent from a Child below 18 will be taken from parent or guardian. It would be better to include all patients irrespective of age. Why children will be excluded is not clear.

 Duration of study was 12 months with 9 months retrospective and three months prospective is a concern as the data for disability should be on a prior fixed interval from bite or else one may underestimate some sequelae. It should be clearly mentioned which time point for detection of sequelae following bite will be used. There are distinct two segments of the study- epidemiological and economic aspects- should be clarified. 

 Snakebite is not 'poisoning' which should be corrected.

 Data analytical plan to be provided.

 Disability: it should be clearly mentioned which group of patients will be interviewed retrospective or prospective or both.

 For the data to be collected in the Case record form there should be a check list and definition for uniformity in data collection with special note on the elements of costing.

 How the psychological disability will be identified is not clear.

 Informed consent: The consent form will be only in English or both in English and local language. For children, assent from will be required.

 Annex 2: Clarity is needed in many fields, for example, how the species of snake will be identified, some fields are vague, example- localized reaction.

 Definition is required in some files, for example adverse reaction.

Is the study design appropriate for the research question?

Partly

Is the rationale for, and objectives of, the study clearly described?

Partly

Are sufficient details of the methods provided to allow replication by others?

No

Are the datasets clearly presented in a useable and accessible format?

Partly

Reviewer Expertise:

Clinician with special interest in snakebite, poisoning and infectious diseases

I confirm that I have read this submission and believe that I have an appropriate level of expertise to confirm that it is of an acceptable scientific standard, however I have significant reservations, as outlined above.

# Response

**Published:** 2021-05-27

**Authors:** Jaideep Menon

**Affiliations:** Amrita Institute of Medical Sciences, India

DR M Abul Faiz

**Abul M Faiz**

 Snakebite is labelled as 'injuries' which is not entirely true. This research will be used for further research and policy formulation, instead may be stated as utilized for policy decision and further research.

 Title: aim and objectives may be mentioned/ changed as  'Objectives with division: General objective (s), and specific objectives.

 Response: Our study has 3 primary objectives i.e. estimating epidemiological and economic burden and community-derived disability weights. Hence we are not reporting as general and specific objectives.

 Being an emergency situation snakebite attending at OPD is unlikely and to be replaced by emergency department or in-patient department, also better to include any victims of snakebite.

 Response: The DALY study is planned in patients treated for snakebite in the immediate three months prior to the start of the study, the assessment for which would occur on follow-up visit as an out-patient. 

 Study design: some contradictory statement are there: adults but mentioned that consent from a Child below 18 will be taken from parent or guardian. It would be better to include all patients irrespective of age. Why children will be excluded is not clear.

 Response: We have now added that response from the child along with those from adult parents or care-givers will be conducted.

 Duration of study was 12 months with 9 months retrospective and three months prospective is a concern as the data for disability should be on a prior fixed interval from bite or else one may underestimate some sequelae. It should be clearly mentioned which time point for detection of sequelae following bite will be used. There are distinct two segments of the study- epidemiological and economic aspects- should be clarified.

 Response: The estimation of epidemiological and economic components will be taken for 12 months duration to estimate annual incidence and economic costs. For the estimation of community-derived disability weights we have used 3 months duration in order to capture the hospitalisation details along with short-term disability aspects. We feel that using a longer time period would induce recall bias of short-term disability by the respondents.

 Snakebite is not 'poisoning' which should be corrected.

 Response: We have now corrected to injury other than due to snakebite.

 Data analytical plan to be provided.

 Response: The data analytical plan is mentioned in the Statistical Analysis sections and has detailed all the primary objectives.

 Disability: it should be clearly mentioned which group of patients will be interviewed retrospective or prospective or both.

 Response: All snakebite victims will be identified who have been bitten in the past. Patient identification is retrospective in nature.

 For the data to be collected in the Case record form there should be a check list and definition for uniformity in data collection with special note on the elements of costing.

 Response: We have used cost of illness checklist of Larg& Moss to capture cost elements.

 How the psychological disability will be identified is not clear.

 Response: Vignettes (pictorial) are used for both cognitive, anxiety and depression in line with the Euro-QoL methods. The victim identifies her level of distress from the vignettes presented and not on the basis of any other formal mental health scoring system.

 Informed consent: The consent form will be only in English or both in English and local language. For children, assent from will be required.

 Response: We will be using consent form in English or Malayalam as understood by the respondent. We have now added that an assent form for children will be used.

 Annex 2: Clarity is needed in many fields, for example, how the species of snake will be identified, some fields are vague, example- localized reaction.

 Response: Species would be reported only in case of the victim being absolutely sure of the species or if the killed/captured snake was verified and reported so.

 Local signs of envenomation limited to the bite site is what is intended in the Questionnaire.

 Definition is required in some files, for example adverse reaction.

 Response: The field officers responsible for entering data would be trained on entry and possible responses. The initial entries are done under supervision of one of the investigators.
